# Reproductive and Developmental Effects of Sex-Specific Chronic Exposure to Dietary Arsenic in Zebrafish (*Danio rerio*)

**DOI:** 10.3390/toxics12040302

**Published:** 2024-04-19

**Authors:** Mahesh Rachamalla, Arash Salahinejad, Vladimir Kodzhahinchev, Som Niyogi

**Affiliations:** 1 Department of Biology, University of Saskatchewan, Saskatoon, SK S7N 5E2, Canada; arash.salahinejad@usask.ca (A.S.); vladimir.kod@usask.ca (V.K.); som.niyogi@usask.ca (S.N.); 2 Toxicology Centre, University of Saskatchewan, 44 Campus Drive, Saskatoon, SK S7N 5B3, Canada

**Keywords:** arsenic, reproductive and developmental toxicity, dietary exposure, HPG-L axis, sex-specific effects

## Abstract

The present study investigated the reproductive and developmental effects of sex-specific chronic exposure to dietary arsenic in zebrafish. Adult zebrafish (*Danio rerio*) were exposed to environmentally realistic doses of arsenic via diet [0 (control; no added arsenic), 30 (low), 60 (medium), and 100 (high) μg/g dry weight, as arsenite] for 90 days. Following exposure, arsenic-exposed females from each dietary treatment were mated with control males, and similarly, arsenic-exposed males from each dietary treatment were mated with control females. In females, arsenic exposure resulted in a dose-dependent decrease in reproductive performance (fecundity, fertilization success, and hatching success). Moreover, a dose-dependent increase in developmental toxicity (larval deformities and larval mortality) was observed with maternal exposure to arsenic. In contrast, in males, arsenic exposure also induced similar reproductive and developmental toxicity; however, the adverse effects were mainly evident only in the medium and high dietary arsenic treatment groups. We also examined the sex-specific effects of dietary arsenic exposure on the expression of genes that regulate the hypothalamus–pituitary–gonadal–liver (HPG-L) axis in fish. The gene expression results indicated the downregulation of HPG-L axis genes in females irrespective of the arsenic treatment dose; however, the reduced expression of HPG-L axis genes in males was recorded only in the medium and high arsenic treatment groups. These observations suggest that chronic arsenic exposure in either females or males causes reproductive and developmental toxicity in zebrafish. However, these toxic effects are markedly higher in females than in males. Our results also suggest that arsenic can act as an endocrine disruptor and mediate reproductive and developmental toxicity by disrupting the HPG-L axis in zebrafish.

## 1. Introduction

Arsenic is a non-essential element that is toxic to a wide range of organisms, including fish. The contamination of arsenic occurs from various anthropogenic sources, including mining and smelting, fossil fuel explorations, hydrological changes, and its use in pesticide products [1]. Arsenic occupies the top position on the list of priority pollutants in the environmental domain maintained by the Agency for Toxic Substances and Disease Registry [2]. Arsenic is also considered the environmental contaminant of highest concern by the World Health Organization [3,4]. These reports suggest that arsenic contamination poses serious risks to human and environmental health [5,6,7].

Arsenic is known to impair several physiological functions in fish, including growth, immune functions, neurodevelopment, ion homeostasis, reproduction, and development [8,9]. However, most of this knowledge of arsenic toxicity is based on studies that examined the effects of arsenic only via waterborne exposure, and much less is known about the adverse effects of dietary arsenic exposure in fish. It is important to note that diet can also be an important source of arsenic to fish inhabiting arsenic-contaminated aquatic ecosystems, where various prey species of fish exhibit elevated arsenic body burden resulting in increased arsenic uptake and accumulation by fish via dietary exposure [10]. Moreover, it has been suggested that fish are more sensitive to arsenic during dietary exposure than waterborne exposure [11]. Although arsenic can exist in both inorganic and organic chemical forms in aquatic ecosystems [12], the inorganic forms (e.g., arsenite and arsenate) are believed to be more toxic to aquatic organisms than the organic forms (e.g., methylarsonate and dimethylarsinate) [13,14]. Interestingly, polychaete worms (*Nereis diversicolor*), a benthic prey species of fish, collected from a mining-impacted creek were found to contain up to 60% arsenic content in their bodies (approximately 80 μg/g dry weight) in inorganic forms (arsenite and arsenate) [15]. This suggests that diet can be a significant source of exposure to highly toxic inorganic forms of arsenic in fish living in contaminated natural waters.

The reproductive effects of arsenic exposure are well documented in mammalian species. Studies conducted with rodents suggest that exposure to inorganic arsenic (e.g., arsenite) impairs reproductive functions in both males and females. Effects in males include a reduction in testicular weight, an imbalance in circulating male sex hormones, and impairment of spermatogenesis and sperm development, leading to low sperm count [16,17]. On the other hand, effects in females include the reduction in circulating female sex hormones, the suppression of ovarian steroidogenesis, prolonged diestrus, the degeneration of reproductive cells (ovarian, follicular, and uterine cells), and an increase in meiotic aberrations in oocytes [18,19]. Unlike in mammals, very few studies have investigated the reproductive and developmental impacts of arsenic exposure in fish, especially under environmentally relevant exposure regimes. Chronic waterborne exposure to arsenite has been reported to cause reduced liver and gonad weight and shortened gonopodia in male fish [20]. In female fish, fecundity (egg production) was one of the most sensitive endpoints during chronic waterborne exposure to arsenite [9]. Chronic ingestion of arsenic-contaminated polychaete worms was similarly found to decrease egg production and spawning frequency in female fish [15].

The physiological and molecular mechanisms by which arsenic causes reproductive toxicity in fish remain largely unknown. Mammalian studies indicate that arsenic-induced oxidative damage in reproductive tissues significantly mediates reproductive toxicity [21,22,23]. Mammalian studies also suggest that arsenic is a potent endocrine disruptor and induces reproductive toxicity by disrupting the endocrine axis (hypothalamus–pituitary–gonad (HPG) axis) that regulates reproductive functions [16,24,25]. A few previous studies conducted in fish also indicated the endocrine-disrupting effects of arsenic. For example, an *in vitro* study using a fish testicular culture system suggested that arsenic inhibits the synthesis of the androgenic sex hormone (11-ketotesterone), leading to decreased spermatogenesis in males [26]. In female oviparous vertebrates like fish, reproduction is regulated by the HPG-L (hypothalamus–pituitary–gonad–liver) axis, as the liver plays a primary reproductive function by synthesizing and secreting vitellogenin under estrogen stimulation [27]. Vitellogenin (*Vtg*) is a lipoglycophosphoprotein absorbed by the growing oocytes in the ovary, and it serves as a major precursor of egg yolk proteins that are essential nutrients for future embryogenesis [28]. Chronic exposure to arsenic-contaminated polychaete worms was found to suppress the expression of the hepatic Vtg gene in female fish, indicating that arsenic can disrupt Vtg production by the liver [15]. Although these observations suggest that arsenic likely causes reproductive toxicity in fish by disrupting the endocrine regulation of reproductive functions in both males and females, the sex-specific effects of arsenic on the whole HPG-L axis and reproductive functions in fish have not been investigated before.

Mammalian studies also reported developmental toxicity due to arsenic, such as delayed development, altered behavior, and fetal malformations following maternal exposure to arsenite [29,30,31]. In fish, direct embryonic exposure to waterborne arsenite induced developmental toxicity in fish, specifically delayed hatching, decreased larval survival, and increased larval deformities [32,33,34]. However, the developmental consequences of parental exposure (maternal or paternal) to arsenic on early development in fish are currently unknown. It is important to note that arsenic can be transferred from the mother to the eggs via the yolk in oviparous animals [35,36], leading to developmental toxicity in fish. Moreover, parental exposure to arsenic can also cause epigenetic alterations [37], which may affect developmental programming, leading to larval toxicity.

With this background in mind, the present study was designed to understand how chronic exposure to environmentally relevant arsenic exposure via diet affects reproductive performance and early development in fish using zebrafish (*Dania rerio*) as a model species. The specific objectives of this study were (i) to characterize how maternal or paternal exposure to dietary arsenic affects reproductive performance (egg production and fertilization rate) and early development (rate of hatching success, larval survival, and deformity) and (ii) to elucidate how dietary arsenic exposure affects the expression of HPG-L axis genes in male and female fish. We hypothesized that maternal and paternal chronic exposure to dietary arsenic would impair zebrafish’s reproductive capacity and larval development in a dose-dependent manner.

## 2. Materials and Methods

### 2.1. Fish Maintenance

Wild-type zebrafish, approximately 6 months old, were procured from the zebrafish stock maintained at the Collaborative Science and Research Building (CSRB) of the University of Saskatchewan (U of S) and housed in the CSRB vivarium. In the current study, a total of 320 adult zebrafish, comprising both sexes, were distributed randomly among 27 2.8 L aquaria, each accommodating approximately 12 fish. The aquaria were supplied with dechlorinated water from the Saskatoon municipal source. The water chemistry parameters were monitored once a week during the entire study period and found to have a consistent pH of 7.6–7.8, a total hardness of 150 mg/L (as CaCO_3_), and an alkalinity of 120 mg/L (as CaCO_3_). Fish were maintained under a photoperiod of 14 h light and 10 h dark and a 25–27 °C temperature. Before their use in the experimental exposures, fish were fed twice daily, with a morning ration of frozen blood worms and an afternoon ration of dried flake food (Nutrafin Max Tropical Fish Flakes, Hagen Deutschland GmbH & Co, Holm, Germany), equivalent to 5% of their body weight. The experimental protocol of the present study was approved by the University of Saskatchewan Animal Research Ethics Board (protocol no. 20190004).

### 2.2. Arsenic-Contaminated Diet Preparation and Experimental Exposure

In this study, fish were treated with 4 different nominal doses of dietary arsenic [0 (control, no added arsenic), 30, 60, and 100 µg/g dry weight (dw)] for 90 days using a flow-through Zebtec Stand-Alone Toxicology Rack system (Tecniplast, Buguggiate, Italy), which carried out the complete exchange of exposure water every hour. The arsenic-contaminated diet was prepared with sodium arsenite (NaAsO_2_; purity > 98%, Sigma-Aldrich, Saint Louis, MO, USA), which was initially dissolved in deionized water and then combined with NutrafinMax Tropical Fish Flakes (Germany). Subsequently, the mixture underwent freeze drying for 48 h in a Labconco freeze dryer. Measurement of the arsenic concentrations in the experimental diets was conducted by inductively coupled plasma mass spectrometry (ICP-MS), as described later. The measured concentrations of arsenic in the diets used in this study were as follows: 1.2 ± 0.3 µg/g dw (control), 38.5 ± 1.2 µg/g dw, 71 ± 3.3 µg/g dw, and 111 ± 4.1 µg/g dw (*n* = 3 for each). The selection of dietary arsenic concentrations in this study was based on a previous investigation, which reported an approximate level of 80 μg/g dw of inorganic arsenic (arsenite and arsenate) in polychaete worms, a prey species of fish, collected from a natural waterbody affected by metal-mining discharge [16]. Thus, the dietary arsenic levels used in our study reflect an environmentally relevant range of arsenic exposure to fish.

Fish were kept in replicate tanks, with different numbers of replicate tanks for each treatment group. The control group consisted of 5 replicate tanks with 60 fish (12 fish per replicate). The dietary treatment groups of 30 µg/g dw and 60 µg/g dw arsenic comprised 7 tanks each, housing 84 fish in each treatment (12 fish per tank). Furthermore, the dietary treatment group of 100 µg/g dw arsenic consisted of eight replicate tanks housing a total of 92 fish (10–12 fish per tank). We maintained a greater number of fish and replicates in the arsenic treatment groups compared to the control group because we expected increased mortality of fish treated with dietary arsenic over 90 days. During the experimental exposure to arsenic, fish were fed with the experimental diet twice a day for 1 h (at a 2.5% body weight ratio each time). The unconsumed food was siphoned out of the exposure tanks immediately after each feeding episode. To assess the possibility of arsenic leaching from the diet into the water, 5 mL of water was sampled from a randomly selected tank within each treatment group after an hour of feeding every two weeks and subsequently analyzed for dissolved arsenic concentration by ICP-MS. As reported elsewhere, the dissolved arsenic concentration never exceeded the maximum of 8 µg/L [25]. Fish mortality was monitored and recorded daily during the experimental exposure to dietary arsenic.

### 2.3. Assessment of Reproductive Performance and Developmental Effects

**Figure 1:**
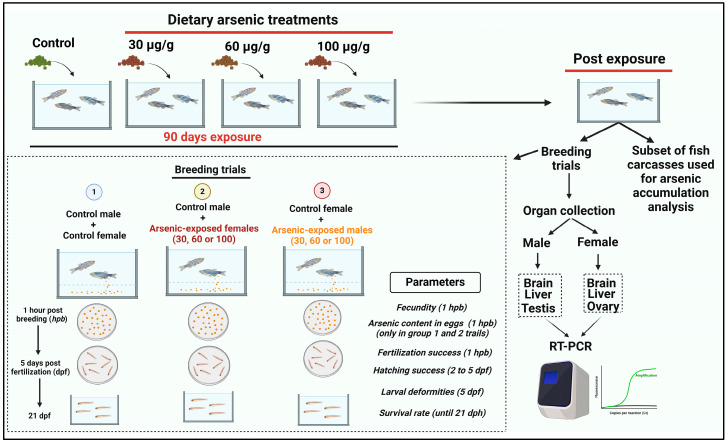
Schematic representation of the experimental design and analysis plan employed in the present study.

The reproductive effects in fish were evaluated after 90 days of dietary arsenic treatment. The design of the dietary arsenic exposures and the subsequent assessments of reproductive and developmental parameters employed in our study are presented in Figure 1. Our objective was to assess how dietary arsenic exposure influences the reproductive performance of zebrafish when either males or females are only exposed to arsenic. Thus, the present study employed the following breeding design: (i) control males X control females, (ii) control males X arsenic-exposed (30, 60, or 100 µg/g dw) females, and (iii) control females X arsenic-exposed (30, 60, or 100 µg/g dw) males. To conduct the breeding trials, breeding trios (1 male and 2 females) were transferred to 2 L breeding tanks, and the male was kept separated from the females overnight using a divider. The breeding tanks were covered overnight with opaque boxes to minimize visual disturbance. The divider was removed in the morning, and the fish were left undisturbed for 2 h for breeding. Subsequently, eggs were collected to evaluate the fecundity and fertilization rate. A subset of eggs produced by the control females and females exposed to different dietary arsenic concentrations was also used to measure the maternal transfer of arsenic to the eggs following the dietary arsenic treatment. Fertilized eggs were placed in E3 medium and reared in Petri dishes until they developed into the swim-up stage. At that point, they were transferred to the holding tanks and reared until they reached the age of 21 days post-hatch (dph). The fertilization rate was evaluated at 1 dpf (day post-fertilization) by calculating the % of live embryos. The hatching success was analyzed at 3–5 dpf by evaluating the % of the ratio of the hatched larvae and the total number of embryos. The larval mortality was monitored and recorded daily for 21 days after hatching.

After completing the breeding trials, adult fish were euthanized using Aquacalm (Metomidate hydrochloride 10 mg/L; Syndel Laboratories, Nanaimo, BC, Canada) and weighed individually. Fish were then dissected and the whole brain, liver, gonad (ovary and testis), and carcass were collected and weighed. The brain, liver, and gonad samples were immediately placed in 5 volumes of RNAlater solution (AM7020, Thermofisher Scientific, Mississauga, ON, Canada) and stored at −80 °C for gene expression analysis later. The carcass samples were stored at −20 °C for further analysis of arsenic accumulation by ICPMS. The gonadosomatic index (GSI) of breeding fish (male or female) was derived by the % of the gonad (testis or ovary) weight (g) and whole-body weight (g).

A subset of 100 larvae from each breeding group was collected at 6 dpf and photographed to evaluate developmental abnormalities. Larvae were anesthetized in a solution of Aquacalm as described previously and carefully placed on a cavity slide coated with 3% methylcellulose. The photographs were taken using a MicroFire^®^ camera (Optronics, Goleta, CA, USA) mounted on a Zeiss Stemi 2000-C Stereo Microscope (Jena, Germany). The photographs were captured using Picture Frame™ Application 2.3 software (Optronics, Tulsa, OK, USA). Multiple pictures were taken of each larva during the imaging process, including the dorsal and lateral views. The morphological development of various anatomical structures was examined using the methods described by Brannen et al. (2010) and Panzica-Kelly et al. (2010) with minor modifications. The analyzed morphological features included body length and shape, tail shape, pectoral fins, heart, swim bladder, abdomen, and craniofacial morphology [38,39]. The rate of larval deformities was expressed as the % of the ratio of larvae with deformities to total living larvae.

### 2.4. Analysis of Arsenic Concentrations

Arsenic concentrations in the water, diet, fish carcasses, and eggs were evaluated by ICP-MS (Agilent 8900, Agilent, Santa Clara, CA, USA) at the Saskatchewan Research Council (SRC) in Saskatoon, Canada. The digestion of experimental diets and fish carcass samples was carried out in 1N HNO_3_ (trace-metal grade, Fisher Scientific, Nepean, ON, Canada) with a 1:5 sample weight to acid volume ratio at a 60° C temperature for 48 h. The digested samples were centrifuged at 15,000× *g* for 5 min, and the supernatants were used for arsenic measurement. Appropriate method blanks, certified arsenic standards (Fisher Scientific, Canada), and a reference material (DOLT-4; National Research Council of Canada) were employed to ensure the quality control and assurance of the arsenic analysis. The recovery of arsenic in the reference material in our analysis was 97%.

### 2.5. Gene Expression Analysis

We measured the expression of the following HPG-L axis genes in zebrafish: *gnrh2* (gonadotropin releasing hormone 2—regulates gonadal development, gametogenesis, and reproductive behavior), *gnrh3* (gonadotropin releasing hormone 3—regulates gonadotropin release, gonadal development, gametogenesis, and reproductive behavior), *fsh-β* (follicle stimulating hormone subunit beta—regulates follicle development and steroidogenesis in the gonads), *lh-β* (luteinizing hormone subunit beta—regulates ovulation, sperm production, and steroidogenesis), *sf-1* (steroidogenic factor-1—regulates steroidogenesis and gonadal development), and *cyp19a1b* (brain aromatase—catalyzes the conversion of androgens to estrogens) in the brain; *fshr* (follicle stimulating hormone receptor—mediates the actions of follicle stimulating hormone), *lhr* (luteinizing hormone receptor—mediates the actions of luteinizing hormone), *sf-1*, *cyp19a1a* (gonadal aromatase—catalyzes the conversion of androgens to estrogens), and *17β-hsd* (*17β*-Hydroxysteroid dehydrogenase—essential for sex steroid synthesis) in gonads; and *Vtg* (vitellogenin—facilitates egg yolk formation and oogenesis) and *ER-α* (estrogen receptor α—mediates the actions of estrogen) in the liver.

The expression of these genes was analyzed by using the real-time quantitative polymerase chain reaction (RT-qPCR) technique. The RNeasy Mini Kit (Qiagen, Hilden, Germany) along with the manufacturer’s recommended DNase treatment was employed for extracting total RNA from the brain, gonad, and liver samples (*n* = 5, with each replicate being a pool of 2–3 respective tissue samples). The extracted RNA was eluted in molecular water and then evaluated for quantity and purity using absorbance ratios at 260 and 280 nm wavelengths measured in a Nanodrop spectrophotometer (NanoDrop, Thermo Scientific, Waltham, MA, USA). cDNA synthesis was performed using 1 μg of total RNA and a QuantiTect Reverse Transcription kit (Qiagen, Germany). RT-qPCR was conducted in triplicate with an iCycler Thermal Cycler (Bio-Rad, Hercules, CA, USA) with a 20 μL reaction mixture comprising 10 μL of SYBR Green PCR Master Mix (Bioline, Taunton, MA, USA), 0.8 μL of each forward and reverse primer, 2 μL of cDNA, and nuclease-free water. The primer sequences (forward and reverse) for each gene were adopted from previous literature (presented in the Supplementary Table S1). The expression of the β-actin gene was used for the normalization of the expression of target genes. The amplification efficiency of all primers ranged from 96% to 103%. The relative quantification of gene expression across different treatment groups was determined using the 2^−ΔΔct^ method [40,41,42,43].

### 2.6. Data Processing and Statistical Analysis

The graphical representations of the data were created using GraphPad prism 10 software developed by Insightful Science, San Diego, CA, USA. SPSS software (version 19, IBM SPSS Inc., Chicago, IL, USA) was used for data processing. Before conducting group comparisons, data were verified for normality of distribution using the Kolmogorov–Smirnov test and for variance homogeneity using Levene’s test. Statistical differences across treatments were then evaluated using one-way ANOVA followed by Dunnett’s post hoc test. Non-parametric data were analyzed using the Kruskal–Wallis and Dunn’s post hoc tests for multiple comparisons. A Kaplan–Meier survival analysis was conducted to analyze the larval survival data, and the Log-rank (Mantel–Cox) test was utilized to identify differences among the treatment groups. Statistical significance was assessed by using a significance level of *p* < 0.05, and the data were expressed as mean ± standard error of the mean (SEM) unless mentioned otherwise.

## 3. Results

### 3.1. Mortality and Arsenic Accumulation in the Tissue and Eggs of Adult Zebrafish Exposed to Chronic Dietary Arsenic

Fish mortality remained <10% in the control and in the 30 µg/g dw and 60 µg/g dw dietary arsenic groups over the 90 days of the exposure period. However, mortality increased significantly (28%; one-way ANOVA, *p* < 0.001) in the 100 µg/g dw dietary arsenic treatment compared to the other treatments. Chronic arsenic exposure via diet resulted in a significant increase in arsenic accumulation in exposed fish compared to the control fish (one-way ANOVA; F_3,16_ = 191.2, *p* < 0.001). Carcass arsenic concentrations in the control and 30 µg/g dw dietary arsenic treatments were 2.2 ± 0.3 and 6.2 ± 2.2 μg/g dw, respectively (mean ± SEM; *n* = 5 for both), which were not significantly different from each other. However, arsenic concentrations in the carcasses from the 60 and 100 μg/g dw dietary arsenic treatments were 29.9 ± 2.5 and 43.3 ± 3.5 μg/g dw, respectively (mean ± SEM, *n* = 5 for both), which were significantly higher relative to the control (Dunnett’s post hoc test: *p* < 0.0001). The carcass arsenic concentrations reported here were analyzed using male and female fish from each dietary arsenic treatment group. Moreover, our results also indicated that chronic exposure to elevated dietary arsenic in females results in the maternal transfer of arsenic to the eggs. The measured arsenic concentrations in the eggs produced by females exposed to 30, 60, and 100 μg/g dw of dietary arsenic were 25 ± 0.7, 102 ± 2.1, and 282 ± 13.8 ng/g dw, respectively, which were all significantly higher than the 0.2 ± 0.02 ng/g dw of arsenic recorded in the eggs produced by control females (mean ± SEM; *n* = 5 for each) (one-way ANOVA followed by Dunnett’s post hoc test, all *p* < 0.001). We did not analyze arsenic levels in the eggs produced by control females that were bred with arsenic-exposed males since no maternal transfer is supposed to occur here, and the transfer of arsenic via sperm, if any, is likely to be insignificant and thus nondetectable.

### 3.2. Reproductive and Developmental Effects of Chronic Arsenic Exposure in Females

**Figure 2:**
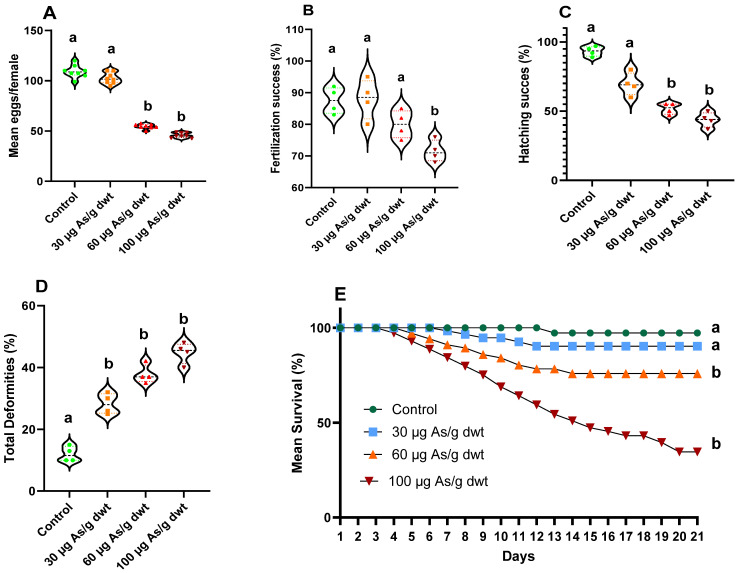
Reproductive and developmental effects of chronic dietary exposure to arsenic in female zebrafish bred with control male zebrafish: (**A**) mean eggs per female [*n* = 8 (each replicate represents a breeding trio: one male and two female)], (**B**) fertilization success (%), (**C**) hatching success (%), (**D**) total larval deformities (%), and (**E**) mean larval survival (%) over 21 days post-hatch. Data presented in plots (**B**–**E**) are averages of 4 replicates (*n* = 4), where each replicate consisted of 100 fertilized eggs. Violin plots show individual data points in different colors and shapes based on the treatment groups, and the width of each plot represents the density of data points along the distribution. The black dotted line in the violin plots indicates the group average, and the different letters above the bars indicate statistically significant differences across different treatment groups (*p* < 0.05).

Here, we describe the results of breeding trials between the control males (not exposed to arsenic) and the females exposed to 0 (control), 30, 60, or 100 μg/g dw of dietary arsenic for 90 days. There was no significance change in the GSI of male and female fish among the four treatment groups. However, the chronic arsenic exposure of females significantly reduced the fecundity (mean eggs produced per female; one-way ANOVA, F_3,28_ = 452.7, *p* < 0.0001) (Figure 2A). Although there was no significant change in fecundity between the control and low (30 μg/g dw) dietary arsenic treatment, a significant reduction (2-fold; Dunnett’s post hoc test: *p* < 0.0001) in fecundity was recorded in females exposed to medium (60 μg/g dw) and high (100 μg/g dw) dietary arsenic compared to the control females. Similarly, chronic dietary arsenic exposure in females also resulted in a significant decrease in fertilization success (%) (Kruskal–Wallis test; X^2^_(3)_ = 10.10, *p* < 0.001) (Figure 2B) and hatching success (%) (Kruskal–Wallis test; X^2^_(3)_ = 13.68, *p* < 0.001) (Figure 2C). A significant reduction in fertilization success (Dunn’s post hoc test: *p* < 0.05) was observed only in females exposed to the high dietary arsenic concentration relative to the control females (Figure 2B). Chronic dietary arsenic exposure in females resulted in a similar dose-dependent effect on hatching success as that observed with fecundity, with a significant reduction in medium and high arsenic treatments relative to the control (Dunn’s post hoc tests: both *p* < 0.05) but no change in the low arsenic treatment (Figure 2C). In addition to the reproductive effects, the total larval deformities (%) were significantly increased (one-way ANOVA F_3,12_ = 100.3, *p* < 0.000) following chronic exposure to dietary arsenic in females (Figure 2D). Several types of developmental deformities, such as swim bladder edema, spinal curvature, and tail deformities, were observed in the offspring following maternal exposure to arsenic (see Figure S1 in the Supplementary Section). The % of larval deformities significantly increased in all the arsenic treatment groups relative to the control (Dunnett’s post hoc tests: all *p* < 0.0001). Finally, the larval survival (%) was significantly decreased following maternal exposure to arsenic (Kaplan–Meier survival analysis: X^2^_3_ = 57.96, *p* < 0.001). The lowest larval survival (~70% decrease) was observed with the high maternal arsenic treatment relative to the control (Log-rank (Mantel–Cox) test: X^2^_1_ = 35.81, *p* < 0.0001), followed by the medium maternal arsenic treatment (~30% decrease) (Log-rank (Mantel-Cox) test: X^2^_1_ = 11.33, *p* = 0.0008), with no significant change in larval survival between the control and low maternal arsenic treatment (Log-rank (Mantel–Cox) test: X^2^_1_ = 2.66, *p* = 0.102) (Figure 2E).

### 3.3. Reproductive and Developmental Effects of Chronic Arsenic Exposure in Males

**Figure 3:**
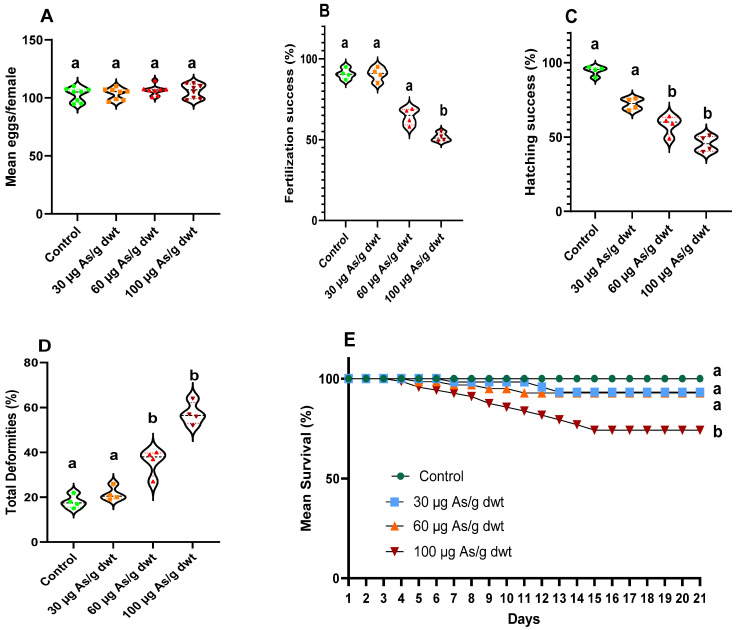
Reproductive and developmental effects of chronic dietary exposure to arsenic in male zebrafish bred with control female zebrafish: (**A**) mean eggs per female [*n* = 8 (each replicate represents a breeding trio: one male and two female)], (**B**) fertilization success (%), (**C**) hatching success (%), (**D**) total larval deformities (%), and (**E**) mean larval survival (%) over 21 days post-hatch. Data presented in plots (**B**–**E**) are averages of 4 replicates (*n* = 4), where each replicate consisted of 100 fertilized eggs. Violin plots show individual data points in different colors and shapes based on the treatment groups, and the width of each plot represents the density of data points along the distribution. The black dotted line in the violin plots indicates the group average, and the different letters above the bars indicate statistically significant differences across different treatment groups (*p* < 0.05).

Here we present the results of breeding trials between the control females (not exposed to arsenic) and the males exposed to 0 (control), 30 (low), 60 (medium), or 100 (high) μg/g dw of dietary arsenic for 90 days. There was no significant change in the GSI of male and female fish among the 4 treatment groups. Similarly, no significant change in fecundity (mean eggs produced per female) was recorded among females used in these breeding trials (one-way ANOVA F_3,28_ = 0.8711, *p* = 0.467, Figure 3A). However, paternal exposure to dietary arsenic significantly reduced the fertilization success (%) (Kruskal–Wallis test; X^2^_(3)_ = 12.61, *p* < 0.0001, Figure 3B) and hatching success (%) (Kruskal–Wallis test; X^2^_(3)_ = 13.66, *p* < 0.0001, Figure 3C). Further post hoc analysis revealed that fertilization success was significantly decreased only in the high paternal arsenic treatment group relative to the control (Dunn’s post hoc test: *p* < 0.01), whereas hatching success was significantly reduced following paternal exposure to medium and high dietary arsenic compared to the control (Dunn’s post hoc test: *p* < 0.05 and <0.01, respectively). Moreover, paternal exposure to arsenic also significantly altered total larval deformities (%) (one-way ANOVA F_3,12_ = 66.30, *p* < 0.0001) and larval survival (Kaplan–Meier survival analysis: X^2^_3_ = 21.21, *p* < 0.0001). A significant increase in larval deformities (combination of same deformity types as described above for maternal arsenic exposure; Figure S1) was observed in the medium and high paternal arsenic treatment groups relative to the control (Dunn’s post hoc test: both *p* < 0.001; Figure 3D). In contrast, a significant decrease (~30%) in larval survival was observed only in the high paternal arsenic treatment group compared to the control (Log-rank (Mantel–Cox) test: X^2^_1_ = 14.03, *p* = 0.0002) (Figure 3E).

### 3.4. Sex-Specific Effects of Chronic Dietary Arsenic Exposure on the Expression of HPG-L Axis Genes in Zebrafish

**Figure 4:**
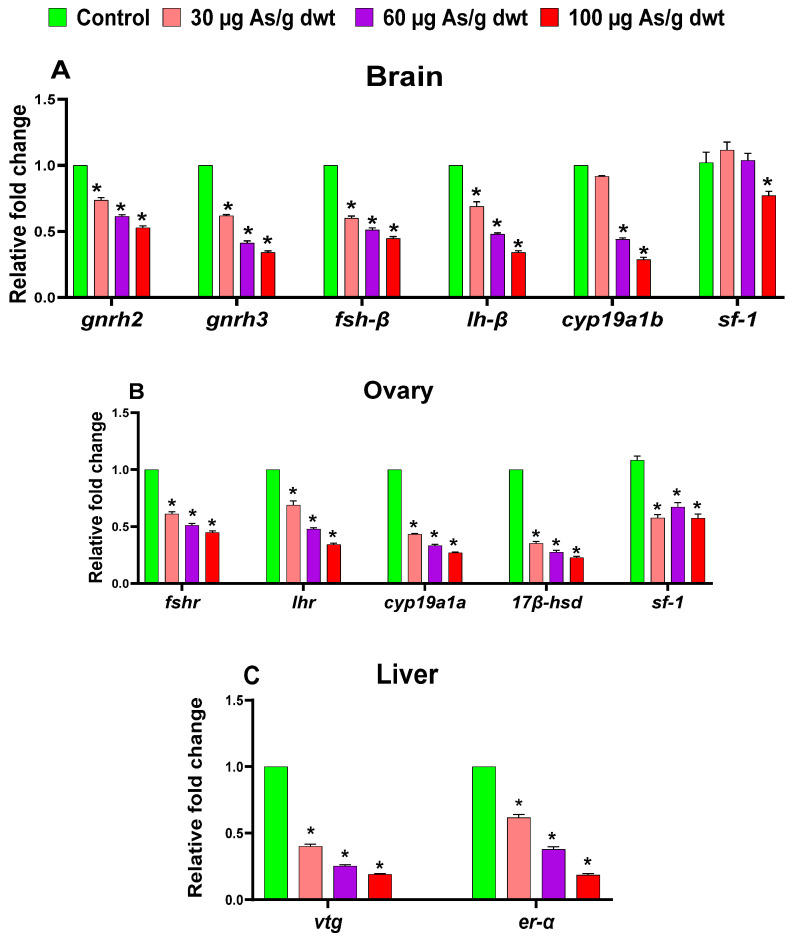
Relative mean fold change in the expression of different HPG-L axis genes in the brain (**A**), ovary (**B**), and liver (**C**) of female zebrafish across different chronic dietary arsenic treatment groups. Data are shown as the mean ± SEM (*n* = 5), and a significant difference (*p* < 0.05) compared to the control is indicated by an asterisk (*).

The one-way ANOVA showed that chronic exposure to arsenic resulted in significant reductions in the expression of all the HPG-L axis genes examined in females, including *gnrh2* (F_3,16_ = 200.5, *p* < 0.0001), *gnrh3* (F_3,16_ = 148, *p* < 0.0001), *fsh-β* (F_3,16_ = 273, *p* < 0.0001), *lh-β* (F_3,16_ = 230.3, *p* < 0.0001), *cyp19a1b* (F_3,16_ = 175.5, *p* < 0.0001), and *sf-1* (F_3,16_ = 6.45, *p* = 0.0045) in the brain (Figure 4A); *fshr* (F_3,16_ = 235, *p* < 0.0001), *lhr* (F_3,16_ = 216, *p* < 0.0001), *cyp19a1a* (F_3,16_ = 213.6, *p* < 0.0001), *17β-hsd* (F_3,16_ = 162.6, *p* < 0.0001), and *sf-1* (F_3,16_ = 45.02, *p* < 0.0001) in the ovary (Figure 4B); and *Vtg* (F_3,16_ = 527.7, *p* < 0.0001) and *ER-α* (F_3,16_ = 282.2, *p* < 0.0001) in the liver (Figure 4C). Subsequently, Dunnett’s post hoc analysis indicated that the expression of several genes (*gnrh2*, *gnrh3*, *fsh-β*, *lh-β*, *fshr*, *lhr*, *cyp19a1a*, *17 β-hsd*, *Vtg*, *er-α*, and *sf-1* (ovary)) was significantly downregulated in arsenic-exposed females, irrespective of arsenic concentrations in the diet, relative to the control females (all *p* < 0.05). In contrast, the expression of the other two genes (*cyp19a1b* and *sf-1* (brain)) was significantly downregulated in females exposed only to medium or high dietary arsenic doses compared to the control females (all *p* < 0.05).

**Figure 5:**
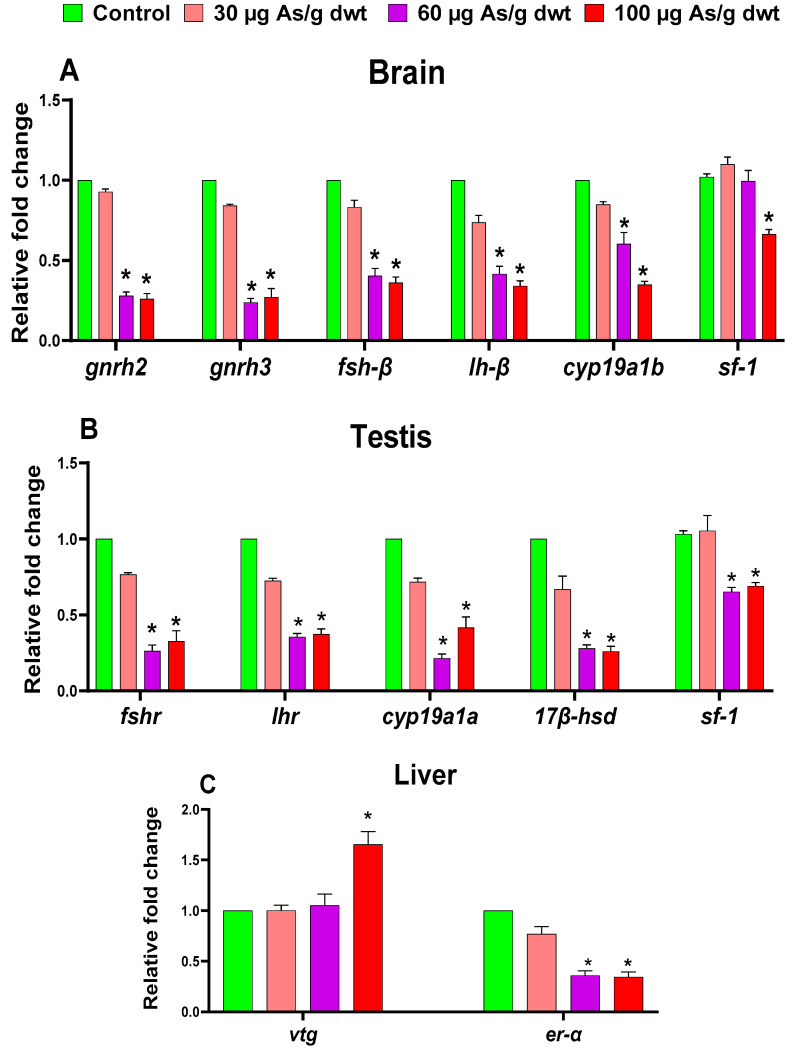
Relative mean fold change in the expression of different HPG-L axis genes in the brain (**A**), testis (**B**), and liver (**C**) of male zebrafish across different chronic dietary arsenic treatment groups. Data are shown as the mean ± SEM (*n* = 5), and a significant difference (*p* < 0.05) compared to the control is indicated by an asterisk (*).

Similarly, the one-way ANOVA also indicated that chronic dietary arsenic exposure resulted in significant alterations in the expression of all the HPG-L axis genes examined in males, including *gnrh2* (F_3,16_ = 722.6, *p* < 0.0001), *gnrh3* (F_3,16_ = 350.9, *p* < 0.0001), *fsh-β* (F_3,16_ = 150, *p* < 0.0001), *lh-β* (F_3,16_ = 191.2, *p* < 0.0001), *cyp19a1b* (F_3,16_ = 113.3, *p* < 0.0001), and *sf-1* (F_3,16_ = 19.64, *p* < 0.0001) in the brain (Figure 5A); *fshr* (F_3,16_ = 222, *p* < 0.0001), *lhr* (F_3,16_ = 119.3, *p* < 0.0001), *Cyp19a1a* (F_3,16_ = 248, *p* < 0.0001), *17β-hsd* (F_3,16_ = 320, *p* < 0.0001), and *sf-1* (F_3,16_ = 15.63, *p* < 0.0001) in the testis (Figure 5B); and *Vtg* (F_3,16_ = 11.69, *p* = 0.0003) and *er-α* (F_3,16_ = 265.4, *p* < 0.0001) in the liver (Figure 5C). Subsequently, Dunnett’s post hoc analysis showed that the expression of all the HPG-L axis genes, except the brain *sf-1* and hepatic *Vtg* genes, which were significantly dysregulated only in the high dietary arsenic treatment group, was downregulated in males exposed to medium and high dietary arsenic doses compared to the control males (all *p* < 0.05). In contrast to females, no change in the expression of any of HPG-L axis genes was recorded in males exposed to low dietary arsenic. Interestingly, the expression of the hepatic *Vtg* gene was significantly upregulated (1.7-fold; *p* < 0.05) in males exposed to high dietary arsenic relative to the control males, which was opposite to the change in *Vtg* gene expression observed in arsenic-exposed females.

## 4. Discussion

To our knowledge, the current study is the first to report sex-specific reproductive and developmental effects of chronic arsenic exposure in any fish species. The observations in the present study demonstrated that environmentally realistic chronic exposure to dietary arsenic in either male or female zebrafish impairs their reproductive performance and induces developmental toxicity in the offspring in a dose-dependent manner. However, the magnitude of the reproductive and developmental effects of chronic arsenic exposure is much higher in females than in males. In addition, the present study also demonstrated that chronic dietary arsenic exposure markedly alters the expression of genes involved in the HPG-L axis in both male and female zebrafish, thereby indicating that the arsenic-induced endocrine disruption likely plays an important role in mediating its reproductive and developmental toxicity in fish.

We observed a 15- and 20-fold increase in arsenic accumulation in fish following chronic exposure to 60 and 100 µg/g dwt dietary arsenic, respectively. A similar dose-dependent increase in arsenic bioaccumulation was also reported previously in zebrafish treated with an arsenic-contaminated natural diet (polychaete worm, *Nereis diversicolor*) [16] and in lake whitefish (*Coregonus clupeaformis*) and lake trout (*Salvelinus namaycush*) treated with dietary arsenic (100–1000 μg/g dwt) for 20 days [44]. Moreover, we recorded a dose-dependent (~125–300-fold) increase in the arsenic burden in the eggs produced by female zebrafish chronically exposed to 30–100 µg/g dwt of arsenic relative to the eggs produced by the control females. This is consistent with previous studies that reported maternal arsenic transfer to the eggs in birds such as black-tailed gull (*Larus crassirostris*) and zebrafish following exposure to elevated arsenic [16,45].

The reproductive and developmental effects of chronic dietary arsenic exposure in males and females were examined separately in our study. When arsenic-exposed females were crossed with control males, it resulted in a 50–60% decrease in fish fecundity (mean eggs/female) and hatching success in the medium (60 µg/g dwt) and high (100 µg/g dwt) arsenic exposure groups, although a significant decrease (~20%) in fertilization success was recorded only in the high arsenic exposure group. On the other hand, when arsenic-exposed males were crossed with control females, we observed a 40–50% decrease in hatching success in the medium and high arsenic treatment groups and a significant (~30%) reduction in fertilization success in the high arsenic treatment group. These observations indicate that chronic dietary arsenic exposure in females or males leads to decreased reproductive performance in zebrafish. Consistent with our observations, Boyle et al. (2008) previously reported decreased fecundity, hatching success, and spawning frequency in zebrafish chronically treated with a field-collected natural diet (polychaete worm) that contained elevated levels of arsenic [16].

Furthermore, our study provided important new insights into the developmental effects of parental arsenic exposure in fish. We observed a significant increase in larval deformities and mortality following maternal and paternal exposure to chronic dietary arsenic in zebrafish. Maternal exposure to arsenic increased the rate of larval deformities by 2–3-fold, irrespective of the arsenic concentration in the diet relative to the control. Maternal exposure to medium and high dietary arsenic doses also induced a ~30–70% increase in larval mortality. Interestingly, paternal exposure to dietary arsenic also increased larval deformity and larval mortality rates. However, the magnitude of these effects was more modest relative to that observed with maternal exposure to arsenic. For example, the rate of larval deformities increased by 1.5–2-fold in the medium and high paternal arsenic treatment groups relative to the control, with no change in the low arsenic treatment group. Moreover, a significant decrease (~30%) in the larval survival rate was only observed in the high paternal arsenic treatment group relative to the control. Nevertheless, our study showed that maternal and paternal arsenic exposure induces similar types of larval deformities, namely spinal curvature and tail deformities and swim bladder edema.

The effects of chronic arsenic exposure, either via water or diet, on the HPG-L axis of fish are largely unknown, and our study is the first to shed light on the endocrine-disrupting effects of arsenic in fish. Our results indicated that chronic dietary arsenic exposure leads to significant alterations in the expression of all the HPG-L axis genes examined in both male and female zebrafish. The arsenic-induced disruption of the expression of HPG-L genes was more pronounced in females than males, as the alterations occurred in females irrespective of the dietary arsenic doses used in our study. In contrast, the alteration in HPG-L axis gene expressions was recorded in males treated with medium and high dietary arsenic doses. Nevertheless, arsenic appears to have broad and sex-specific effects across the whole HPG-L axis—the primary endocrine axis that governs the reproductive functions in teleost fish such as zebrafish.

The HPG-L axis involves three major anatomical components—the brain (hypothalamic and pituitary components), gonads (testes in males and ovaries in females), and the liver. The gonadotropin-releasing hormones (variants *Gnrh2* and *Gnrh3*) secreted by the hypothalamus orchestrate the secretion of gonadotropins, namely follicle-stimulating hormone (FSH) and luteinizing hormone (LH), from the pituitary gland [46,47]. These gonadotropins are heterodimeric glycoproteins that are characterized by a common subunit known as the glycoprotein hormone α subunit (GPα) and specific subunits, *Fshβ* and *Lhβ* [48]. FSH and LH exert their functions by binding to their specific gonadal receptors (*Fshr* and *Lhr*, respectively). Specifically, these gonadotropins stimulate follicular maturation, oogenesis, ovulation, and the conversion of androgens to estrogens (17β estradiol or E2) in females, whereas they facilitate spermatogenesis and the synthesis of testosterone (T), particularly 11-ketotestosterone (11 KT), in males [49]. In addition, *sf-1*, a nuclear receptor, is also a key regulator of sex steroid synthesis in gonads, but it is also expressed in the brain and modulates the HPG-L axis by regulating the functions of GnRH as well as LH and FSH [50,51]. The aromatase enzymes, gonad-specific *cyp19a1a* and brain-specific *cyp19a1b,* and the gonadal oxidoreductase *17β-hsd* are key regulators of androgen and estrogen synthesis [52]. Furthermore, vitellogenesis is crucial for oogenesis in female teleosts, as *Vtg* constitutes the principal component of egg yolk protein, and the synthesis of *Vtg* in the liver is triggered by the binding of circulating E2 to estrogen receptors (e.g., *ERα*) [53].

The present study showed that arsenic exposure reduced the expression of the *Gnrh2*, *Gnrh3*, *Fshβ*, *Lhβ*, and *sf-1* genes in the brain in both male and female fish. This indicated that arsenic likely disrupts the synthesis or release of gonadotropin-releasing hormones in the hypothalamus irrespective of gender, leading to the downregulation of *Fshβ* and *Lhβ* genes. It is unclear how arsenic disrupts the stimulatory control of the gonadotropic axis by *Gnrh*, as it has not been investigated before in any fish species. Nonetheless, dopamine regulates reproductive functions at different levels along the HPG-L axis in fish, and dopamine has been suggested to specifically inhibit the expression of *Gnrh*, *Fshβ*, and *Lhβ* in female zebrafish brains [54,55]. Our previous study demonstrated that chronic dietary exposure to arsenic (30–100 μg/g dwt) increases the dopamine level in zebrafish brains [25]; thus, the increased dopamine level in the brain might have contributed to the downregulation of gonadotropic axis genes observed in the present study. Our observations are also consistent with mammalian studies where chronic exposure to arsenic has been found to reduce the circulating levels of gonadotropins (FSH and LH) and impaired reproductive functions in both males and females [23,56,57,58].

Moreover, the decrease in the expression of aromatase (*cyp19a1b* in the brain and *cyp19a1a* in the gonads) and *17β-hsd* genes in males and females in our study might have occurred due to the downregulation of brain and gonadal *sf-1*, which is known to regulate the activity of these steroidogenic enzymes [59]. It has been suggested that *sf-1* plays a crucial role in zebrafish reproduction by regulating the differentiation of gonadal tissues and the production of sex steroids in gonads by modulating the expression of genes encoding for enzymes involved in converting cholesterol into various sex steroids, including testosterone and estradiol [60]. Consistent with our observations, arsenic has been found to downregulate the mRNA expression of *sf-1* and the steroidogenic enzymes in the testicular Leydig cells of mice [61,62]. Previous rodent studies also reported reduced expression of the gonadal *17β-hsd* gene and a decrease in the plasma sex steroid levels following chronic treatment with arsenic [26,63,64]. Additionally, we also recorded a decreased expression of gonadotropin receptor genes (*Fshr* and *Lhr*) in the gonads of male and female zebrafish following chronic exposure to arsenic, which might be a consequence of the downregulation of the *Fshβ* and *Lhβ* genes in the brain, as suggested in mammalian studies [65]. Since FSH and LH are key regulators of enzymes in synthesizing androgens and estrogens, it is conceivable, based on our observations, that arsenic likely disrupts steroidogenesis via the downregulation of gonadal *sf-1* and by disrupting the effects of FSH and LH on the testis and ovary of male and female zebrafish, respectively.

The decreased hepatic mRNA expression of *ERα* in both male and female zebrafish observed in the present study indicates that estrogen signaling was disrupted by arsenic exposure, which has also been suggested in rodent studies [58,64,66]. Interestingly, we observed an opposite response in the expression of the hepatic *Vtg* gene between male and female fish following chronic arsenic exposure in the current study. Arsenic was found to suppress hepatic *Vtg* gene expression in females, indicating that arsenic likely disrupts vitellogenin synthesis in the liver, which can be attributed to reduced fecundity in arsenic-exposed females. Decreased expression of the *Vtg* gene has also been previously reported in female zebrafish fed with arsenic-contaminated wild polychaete worms [16]. Unlike in females, chronic exposure to arsenic resulted in an increased expression of the hepatic *Vtg* gene in males, albeit only at the high exposure concentration (100 μg/g dwt), in the present study. Very little is known about the gender-specific effects of arsenic exposure on hepatic *Vtg* expression in oviparous animals, and further studies are required to understand the reproductive implications of increased hepatic *Vtg* expression in male zebrafish, such as feminization.

Although no previous study examined the early developmental effects of parental arsenic exposure via water or diet in fish, direct embryonic exposure to waterborne arsenite, albeit at extremely high concentrations (>65 mg/L), was reported to cause teratogenic effects in zebrafish. For example, embryonic exposure (up to 96–120 h post-fertilization) to arsenite via water was found to decrease the larval survival rate and induce developmental abnormalities such as delayed hatching, growth retardation, and larval deformities including scoliosis and edema in zebrafish [33,34]. Maternal exposure to arsenic is known to cause congenital effects in mammals, including reduced fetal growth, fetal malformation, and even death [67]. The developmental toxicity of maternal arsenic exposure observed in the present study can be attributed to the maternal transfer of arsenic into the eggs, as embryonic arsenic exposure has been suggested to cause developmental toxicity by inducing oxidative stress in zebrafish [33]. However, the developmental effects of paternal exposure to arsenic observed in our study indicate the involvement of other potential mechanisms since there was no maternal transfer of arsenic during this exposure. Mammalian studies suggest arsenic exposure can impair sperm production and sperm quality, likely due to the disruption of the HPG axis, and thereby can ultimately affect embryonic development [68,69,70]. Moreover, parental exposure to arsenic can also affect gonadal genes and early developmental programming by epigenetic mechanisms, such as abnormal DNA methylation patterns, as observed during early embryonic exposure to arsenic in zebrafish [34]. Future studies should focus on understanding the molecular mechanisms by which parental exposure to arsenic causes developmental toxicity in fish.

In conclusion, our study demonstrated that chronic dietary arsenic exposure to male or female zebrafish leads to reproductive impairment and developmental toxicity. We also demonstrated that arsenic is an endocrine disruptor, and it disrupts the HPG-L axis at multiple organ levels irrespective of gender, which is likely a key contributing factor underlying its reproductive and developmental toxicity in zebrafish. However, the precise mechanisms by which arsenic disrupts the HPG-L axis in fish are currently not very well understood. Arsenic probably mediates the disruption of the HPG-L axis in fish by multiple mechanisms, including the disruption of dopaminergic signaling, interactions with hormone receptors, altered DNA methylation, enzymatic dysfunction, and oxidative tissue damage, which should be the focus of future investigations.

## Data Availability

The datasets used and/or analysed during the current study are available from the corresponding author on reasonable request.

## Supplementary Materials

The following supporting information can be downloaded at https://www.mdpi.com/article/10.3390/toxics12040302/s1, Table S1: Sequences of the quantitative PCR primers used for the RT-qPCR analysis in the present study. Figure S1: Representative photographs of morphological deformities observed in the zebrafish larvae produced by the breeding of arsenic-exposed females and control males, and arsenic-exposed males and control females.

## References

[1] Hughes M.F., Beck B.D., Chen Y., Lewis A.S., Thomas D.J. (2011). Arsenic Exposure and Toxicology: A Historical Perspective. Toxicol. Sci..

[2] ATSDR (2007). Toxicological Profile for Arsenic. https://www.atsdr.cdc.gov/toxprofiles/tp2.pdf.

[3] Kumar A., Raj V., Srivastava A., Ali M., Ghosh A.K., Rachamalla M., Kumar D. (2022). Autophagy in Arsenic Exposed Population and Cancer Patients. Autophagy and Metabolism: Potential Target for Cancer Therapy.

[4] Kumar A., Ali M., Raj V., Kumari A., Rachamalla M., Niyogi S., Kumar D., Sharma A., Saxena A., Panjawani G. (2023). Arsenic Causing Gallbladder Cancer Disease in Bihar. Sci. Rep..

[5] Thakur M., Rachamalla M., Niyogi S., Datusalia A.K., Flora S.J.S. (2021). Molecular Mechanism of Arsenic-Induced Neurotoxicity Including Neuronal Dysfunctions. Int. J. Mol. Sci..

[6] Verma N., Rachamalla M., Kumar P.S., Dua K. (2023). Assessment and Impact of Metal Toxicity on Wildlife and Human Health. Metals in Water.

[7] Sharma R., Abubakar M.D., Bisht P., Rachamalla M., Kumar A., Murti K., Ravichandiran V., Kumar N. (2023). Arsenic Exposure and Amyloid Precursor Protein Processing: A Focus on Alzheimer’s Disease. Curr. Mol. Pharmacol..

[8] Jamwal A., Rachamalla M., Niyogi S. (2023). Environmental Toxicology of Arsenic to Wildlife (Nonhuman Species): Exposure, Accumulation, Toxicity, and Regulations. Handbook of Arsenic Toxicology.

[9] Kumari B., Kumar V., Sinha A.K., Ahsan J., Ghosh A.K., Wang H., DeBoeck G. (2017). Toxicology of Arsenic in Fish and Aquatic Systems. Environ. Chem. Lett..

[10] Rachamalla M., Chinthada J., Kushwaha S., Putnala S.K., Sahu C., Jena G., Niyogi S. (2022). Contemporary Comprehensive Review on Arsenic-Induced Male Reproductive Toxicity and Mechanisms of Phytonutrient Intervention. Toxics.

[11] Cottingham K.L., Karimi R., Gruber J.F., Zens M.S., Sayarath V., Folt C.L., Punshon T., Morris J.S., Karagas M.R. (2013). Diet and Toenail Arsenic Concentrations in a New Hampshire Population with Arsenic-Containing Water. Nutr. J..

[12] Erickson R.J., Mount D.R., Highland T.L., Hockett J.R., Hoff D.J., Jenson C.T., Lahren T.J. (2019). The Effects of Arsenic Speciation on Accumulation and Toxicity of Dietborne Arsenic Exposures to Rainbow Trout. Aquat. Toxicol..

[13] Zhang W., Miao A.J., Wang N.X., Li C., Sha J., Jia J., Alessi D.S., Yan B., Ok Y.S. (2022). Arsenic Bioaccumulation and Biotransformation in Aquatic Organisms. Environ. Int..

[14] Geiszinger A.E., Goessler W., Francesconi K.A. (2002). The Marine Polychaete Arenicola Marina: Its Unusual Arsenic Compound Pattern and Its Uptake of Arsenate from Seawater. Mar. Env. Res..

[15] Fattorini D., Regoli F. (2004). Arsenic Speciation in Tissues of the Mediterranean Polychaete *Sabella spallanzanii*. Env. Toxicol. Chem..

[16] Boyle D., Brix K.V., Amlund H., Lundebye A.K., Hogstrand C., Bury N.R. (2008). Natural Arsenic Contaminated Diets Perturb Reproduction in Fish. Env. Sci. Technol..

[17] Choudhury B.P., Roychoudhury S., Sengupta P., Toman R., Dutta S., Kesari K.K. (2022). Arsenic-Induced Sex Hormone Disruption: An Insight into Male Infertility. Advances in Experimental Medicine and Biology.

[18] Flora S.J.S., Agrawal S. (2017). Arsenic, Cadmium, and Lead. Reproductive and Developmental Toxicology.

[19] Navarro P.A.A.S., Liu L., Keefe D.L. (2004). In Vivo Effects of Arsenite on Meiosis, Preimplantation Development, and Apoptosis in the Mouse. Biol. Reprod..

[20] Navarro P.A.A.S., Liu L., Ferriani R.A., Keefe D.L. (2006). Arsenite Induces Aberrations in Meiosis That Can Be Prevented by Coadministration of N-Acetylcysteine in Mice. Fertil. Steril..

[21] Smith R.J., Kollus K.M., Propper C.R. (2022). Environmentally Relevant Arsenic Exposure Affects Morphological and Molecular Endpoints Associated with Reproduction in the Western Mosquitofish, *Gambusia affinis*. Sci. Total Environ..

[22] Erkan M., Aydin Y., Yilmaz B.O., Yildizbayrak N. (2020). Arsenic-Induced Oxidative Stress in Reproductive Systems. Toxicology: Oxidative Stress and Dietary Antioxidants.

[23] Raeeszadeh M., Karimfar B., Amiri A.A., Akbari A. (2021). Protective Effect of Nano-Vitamin C on Infertility Due to Oxidative Stress Induced by Lead and Arsenic in Male Rats. J. Chem..

[24] Zargari F., Rahaman M.S., KazemPour R., Hajirostamlou M. (2022). Arsenic, Oxidative Stress and Reproductive System. J. Xenobiot..

[25] Rachamalla M., Salahinejad A., Khan M., Datusalia A.K., Niyogi S. (2023). Chronic Dietary Exposure to Arsenic at Environmentally Relevant Concentrations Impairs Cognitive Performance in Adult Zebrafish (*Danio rerio*) via Oxidative Stress and Dopaminergic Dysfunction. Sci. Total Environ..

[26] Ijaz M.U., Haider S., Tahir A., Afsar T., Almajwal A., Amor H., Razak S. (2023). Mechanistic Insight into the Protective Effects of Fisetin against Arsenic-Induced Reproductive Toxicity in Male Rats. Sci. Rep..

[27] Celino F.T., Yamaguchi S., Miura C., Miura T. (2009). Arsenic Inhibits in Vitro Spermatogenesis and Induces Germ Cell Apoptosis in Japanese Eel (*Anguilla japonica*). Reproduction.

[28] Tramunt B., Montagner A., Tan N.S., Gourdy P., Rémignon H., Wahli W. (2021). Roles of Estrogens in the Healthy and Diseased Oviparous Vertebrate Liver. Metabolites.

[29] Hara A., Hiramatsu N., Fujita T. (2016). Vitellogenesis and Choriogenesis in Fishes. Fish. Sci..

[30] Desesso J.M. (2001). Teratogen Update: Inorganic Arsenic. Teratology.

[31] Chattopadhyay S., Ghosh D. (2010). The Involvement of Hypophyseal-Gonadal and Hypophyseal-Adrenal Axes in Arsenic-Mediated Ovarian and Uterine Toxicity: Modulation by HCG. J. Biochem. Mol. Toxicol..

[32] Chattopadhyay S., Bhaumik S., Nag Chaudhury A., Das Gupta S. (2002). Arsenic Induced Changes in Growth Development and Apoptosis in Neonatal and Adult Brain Cells in Vivo and in Tissue Culture. Toxicol. Lett..

[33] Adeyemi J.A., Da Cunha Martins A., Barbosa F. (2015). Teratogenicity, Genotoxicity and Oxidative Stress in Zebrafish Embryos (*Danio rerio*) Co-Exposed to Arsenic and Atrazine. Comp. Biochem. Physiol. Part C Toxicol. Pharmacol..

[34] Li X., Ma Y., Li D., Gao X., Li P., Bai N., Luo M., Tan X., Lu C., Ma X. (2012). Arsenic Impairs Embryo Development via Down-Regulating Dvr1 Expression in Zebrafish. Toxicol. Lett..

[35] Sun H.J., Zhang J.Y., Wang Q., Zhu E., Chen W., Lin H., Chen J., Hong H. (2019). Environmentally Relevant Concentrations of Arsenite Induces Developmental Toxicity and Oxidative Responses in the Early Life Stage of Zebrafish. Environ. Pollut..

[36] Kubota R., Kunito T., Tanabe S., Ogi H., Shibata Y. (2002). Maternal Transfer of Arsenic to Eggs of Black-Tailed Gull (*Larus crassirostris*) from Rishiri Island, Japan. Appl. Organomet. Chem..

[37] Ebisuda K.I., Kunito T., Kubota R., Tanabe S. (2002). Arsenic Concentrations and Speciation in the Tissues of Ringed Seals (*Phoca hispida*) from Pangnirtung, Canada. Appl. Organomet. Chem..

[38] Chakraborty A., Ghosh S., Biswas B., Pramanik S., Nriagu J., Bhowmick S. (2022). Epigenetic Modifications from Arsenic Exposure: A Comprehensive Review. Sci. Total Environ..

[39] Panzica-Kelly J.M., Zhang C.X., Danberry T.L., Flood A., DeLan J.W., Brannen K.C., Augustine-Rauch K.A. (2010). Morphological Score Assignment Guidelines for the Dechorionated Zebrafish Teratogenicity Assay. Birth Defects Res. B Dev. Reprod. Toxicol..

[40] Brannen K.C., Panzica-Kelly J.M., Danberry T.L., Augustine-Rauch K.A. (2010). Development of a Zebrafish Embryo Teratogenicity Assay and Quantitative Prediction Model. Birth Defects Res. B Dev. Reprod. Toxicol..

[41] Schmittgen T.D., Livak K.J. (2001). Analysis of Relative Gene Expression Data Using Real-Time Quantitative PCR and the 2(-Delta Delta C(T)) Method. Methods.

[42] Salahinejad A., Attaran A., Meuthen D., Rachamalla M., Chivers D.P., Niyogi S. (2022). Maternal Exposure to Bisphenol S Induces Neuropeptide Signaling Dysfunction and Oxidative Stress in the Brain, and Abnormal Social Behaviors in Zebrafish (*Danio rerio*) Offspring. Sci. Total Environ..

[43] Kodzhahinchev V., Rachamalla M., Al-Dissi A., Niyogi S., Weber L.P. (2023). Examining the Subchronic (28-Day) Effects of Aqueous Cd-BaP Co-Exposure on Detoxification Capacity and Cardiac Function in Adult Zebrafish (*Danio rerio*). Aquat. Toxicol..

[44] Pedlar R.M., Ptashynski M.D., Wautier K.G., Evans R.E., Baron C.L., Klaverkamp J.F. (2002). The Accumulation, Distribution, and Toxicological Effects of Dietary Arsenic Exposure in Lake Whitefish (*Coregonus clupeaformis*) and Lake Trout (*Salvelinus namaycush*). Comp. Biochem. Physiol. C Toxicol. Pharmacol..

[45] Kubota R., Kunito T., Tanabe S. (2002). Chemical Speciation of Arsenic in the Livers of Higher Trophic Marine Animals. Proc. Mar. Pollut. Bull..

[46] Dufour S., Sebert M.E., Weltzien F.A., Rousseau K., Pasqualini C. (2010). Neuroendocrine Control by Dopamine of Teleost Reproduction. J. Fish. Biol..

[47] Simonneaux V., Ancel C., Poirel V.J., Gauer F. (2013). Kisspeptins and RFRP-3 Act in Concert to Synchronize Rodent Reproduction with Seasons. Front. Neurosci..

[48] Pierce J.G., Parsons T.F. (1981). Glycoprotein Hormones: Structure and Function. Annu. Rev. Biochem..

[49] Levavi-Sivan B., Bogerd J., Mañanós E.L., Gómez A., Lareyre J.J. (2010). Perspectives on Fish Gonadotropins and Their Receptors. Gen. Comp. Endocrinol..

[50] Parker K.L., Rice D.A., Lala D.S., Ikeda Y., Luo X., Wong M., Bakke M., Zhao L., Frigeri C., Hanley N.A. (2002). Steroidogenic Factor 1: An Essential Mediator of Endocrine Development. Recent. Prog. Horm. Res..

[51] Hoivik E.A., Lewis A.E., Aumo L., Bakke M. (2010). Molecular Aspects of Steroidogenic Factor 1 (SF-1). Mol. Cell Endocrinol..

[52] Trant J.M., Gavasso S., Ackers J., Chung B.C., Place A.R. (2001). Developmental Expression of Cytochrome P450 Aromatase Genes (CPY19a and CYP19b) in Zebrafish Fry (*Danio rerio*). Proc. J. Exp. Zool..

[53] Polzonetti-Magni A.M., Mosconi G., Soverchia L., Kikuyama S., Carnevali O. (2004). Multihormonal Control of Vitellogenesis in Lower Vertebrates. Int. Rev. Cytol..

[54] Fontaine R., Affaticati P., Yamamoto K., Jolly C., Bureau C., Baloche S., Gonnet F., Vernier P., Dufour S., Pasqualini C. (2013). Dopamine Inhibits Reproduction in Female Zebrafish (*Danio rerio*) via Three Pituitary D2 Receptor Subtypes. Endocrinology.

[55] Barsagade V.G. (2020). Dopamine System in the Fish Brain: A Review on Current Knowledge. J. Entomol. Zool. Stud..

[56] Zubair M., Ahmad M., Qureshi Z.I. (2017). Review on Arsenic-Induced Toxicity in Male Reproductive System and Its Amelioration. Andrologia.

[57] Akram Z., Jalali S., Shami S.A., Ahmad L., Batool S., Kalsoom O. (2010). Adverse Effects of Arsenic Exposure on Uterine Function and Structure in Female Rat. Exp. Toxicol. Pathol..

[58] Chatterjee A., Chatterji U. (2010). Arsenic Abrogates the Estrogen-Signaling Pathway in the Rat Uterus. Reprod. Biol. Endocrinol..

[59] Val P., Lefrançois-Martinez A.M., Veyssière G., Martinez A. (2003). SF-1 a Key Player in the Development and Differentiation of Steroidogenic Tissues. Nucl. Recept..

[60] Roy Moulik S., Pal P., Majumder S., Mallick B., Gupta S., Guha P., Roy S., Mukherjee D. (2016). Gonadotropin and Sf-1 Regulation of Cyp19a1a Gene and Aromatase Activity during Oocyte Development in the Rohu, *L. rohita*. Comp. Biochem. Physiol. A Mol. Integr. Physiol..

[61] Chen Y., Sun Y., Zhao A., Cai X., Yu A., Xu Q., Wang P., Yao J., Wang Q., Wang W. (2022). Arsenic Exposure Diminishes Ovarian Follicular Reserve and Induces Abnormal Steroidogenesis by DNA Methylation. Ecotoxicol. Env. Saf..

[62] Taşçi T., Eldem V., Erkan M. (2019). Sodium Arsenic Alters the Gene Expression of Some Steroidogenic Genes in TM3 Leydig Cell. Celal Bayar Üniversitesi Fen. Bilim. Derg..

[63] Seif M., Abd El-Aziz T., Sayed M., Wang Z. (2021). *Zingiber officinale* Ethanolic Extract Attenuates Oxidative Stress, Steroidogenic Gene Expression Alterations, and Testicular Histopathology Induced by Sodium Arsenite in Male Rats. Environ. Sci. Pollut. Res..

[64] Pandey R., Garg A., Gupta K., Shukla P., Mandrah K., Roy S., Chattopadhyay N., Bandyopadhyay S. (2022). Arsenic Induces Differential Neurotoxicity in Male, Female, and E2-Deficient Females: Comparative Effects on Hippocampal Neurons and Cognition in Adult Rats. Mol. Neurobiol..

[65] Kishi H., Kitahara Y., Imai F., Nakao K., Suwa H. (2018). Expression of the Gonadotropin Receptors during Follicular Development. Reprod. Med. Biol..

[66] Jana K., Jana S., Samanta P.K. (2006). Effects of Chronic Exposure to Sodium Arsenite on Hypothalamo-Pituitary-Testicular Activities in Adult Rats: Possible an Estrogenic Mode of Action. Reprod. Biol. Endocrinol..

[67] Wang A., Holladay S.D., Wolf D.C., Ahmed S.A., Robertson J.L. (2006). Reproductive and Developmental Toxicity of Arsenic in Rodents: A Review. Int. J. Toxicol..

[68] Machado-Neves M. (2022). Effect of Heavy Metals on Epididymal Morphology and Function: An Integrative Review. Chemosphere.

[69] Souza A.C.F., Machado-Neves M., Bastos D.S.S., Couto Santos F., Guimarães Ervilha L.O., Coimbra J.L.d.P., Araújo L.d.S., Oliveira L.L.d., Guimarães S.E.F. (2021). Impact of Prenatal Arsenic Exposure on the Testes and Epididymides of Prepubertal Rats. Chem. Biol. Interact..

[70] Okamura K., Sato M., Suzuki T., Nohara K. (2022). Inorganic Arsenic Exposure-Induced Premature Senescence and Senescence-Associated Secretory Phenotype (SASP) in Human Hepatic Stellate Cells. Toxicol. Appl. Pharmacol..

