# Supplementary material for: Reproductive and Developmental Effects of Sex-Specific Chronic Exposure to Dietary Arsenic in Zebrafish (Danio rerio)
Source: Toxics. 2024 Apr 19;12(4):302. doi: 10.3390/toxics12040302 (PMC11053724; doi:10.3390/toxics12040302)

**Supplementary Table S1:** Sequences of the quantitative PCR primers used for the RT-qPCR analysis in the present study.

| S.No | Gene | sense (5′–3′) | Antisense (5′–3′) |
|---|---|---|---|
| 1 | *gnrh2* | CAGAGGTTTCAGAGGAAGTGAAGC | TGAGGGCATCCAGCAGTATTG |
| 2 | *gnrh3* | TGGAGGCAACATTCAGGATGT | CCACCTCATTCACTATGTGTATTGG |
| 3 | *fsh-β* | GCTGGACAATGGATCGAGTTTA | CTCGTAGCTCTTGTACATCAAGTT |
| 4 | *lh-β* | GGCTGGAAATGGTGTCTTCT | CCACCGATACCGTCTCATTTAC |
| 5 | *cyp19a1b* | ACTAAGCAAGTCCTCCGCTGTGTACC | TTTAAACATACCGATGCATTGCAGACC |
| 6 | *fshr* | CGTCTCTTTTGTGCACTGGA | GTGGCAATTCCACACTTCCT |
| 7 | *lhr* | CCTGGTCGTCCTGCTGGTT | AAGGCTAGATGGCACATTAGAAATC |
| 8 | *cyp19a1a* | AGATGTCGAGTTAAAGATCCTGCA | CGACCGGGTGAAAACGTAGA |
| 9 | *sf-1* | TCAGCTTGGACGTGAAGAAC | AACTTGTCTGTCTGCTGAGG |
| 10 | *17β-hsd* | ACATTCACGGCTGAGGAGTTT | ATGCTGCCATACGTTTGGTC |
| 11 | *vtg* | AGATCGGTACTTGGCACACC | TGTCGTTTTCAGCAATGGAG |
| 12 | *er-α* | GGTCCAGTGTGGTGTCCTCT | CACACGACCAGACTCCGTAA |
| 13 | *β-actin* | AGGTCATCACCATTGGCAAT | GATGTCCACGTCGCACTTCAT |

**Supplementary Figure S1.** Representative photographs of morphological deformities observed in the zebrafish larvae produced by the breeding of arsenic-exposed females and control males, and arsenic-exposed males and control females.

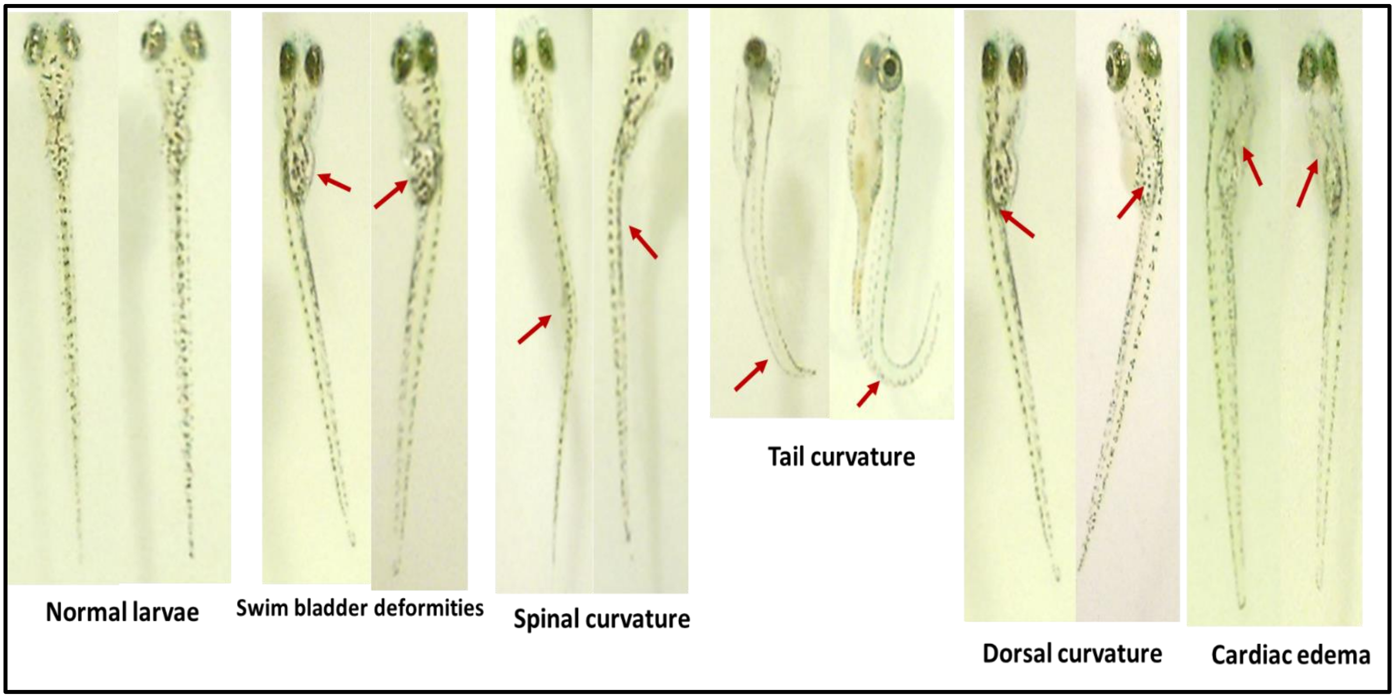

Supplement: Supplementary file 1 [file toxics-12-00302-s001.zip › toxics-2952303-supplementary.pdf]
